# HER2-low status as a distinct breast cancer subtype: myth or truth? Analysis of the WSG trials WSG-ADAPT-HR+/HER2-, WSG-PlanB, and WSG-ADAPT-TN

**DOI:** 10.1186/s13058-025-01969-z

**Published:** 2025-02-14

**Authors:** Gilda Schmidt, Oleg Gluz, Matthias Christgen, Mattea Reinisch, Sherko Kümmel, Ulrike Nitz, Michael Braun, Bahriye Aktas, Kerstin Lüdtke-Heckenkamp, Helmut Forstbauer, Eva-Maria Grischke, Claudia Schumacher, Rolf Mahlberg, Wolfram Malter, Toralf Reimer, Benno Nuding, Andrea Stefek, Rachel Wuerstlein, Monika Graeser, Katarzyna Jóźwiak, Sandy Burmeister, Christine zu Eulenburg, Michael Lauseker, Cornelia Kolberg-Liedtke, Aleix Prat, Peter Schmid, Rick Baehner, Hans Heinrich Kreipe, Erich-Franz Solomayer, Nadia Harbeck, on behalf of the West German Stud y Group investigators

**Affiliations:** 1 https://ror.org/01jdpyv68 grid.11749.3a 0000 0001 2167 7588 Department of Gynecology, Obstetrics and Reproductive Medicine, Saarland University Medical Center, Homburg, Germany; 2 https://ror.org/040ypnf94 grid.476830.e West German Study Group, Moenchengladbach, Germany; 3 https://ror.org/037hxym60 grid.440216.5 0000 0004 0415 9393 Breast Center Niederrhein, Ev. Hospital Bethesda, Moenchengladbach, Germany; 4 University Clinics Cologne, Cologne, Germany; 5 https://ror.org/00f2yqf98 grid.10423.34 0000 0000 9529 9877 Institute of Pathology, Hannover Medical School, Hannover, Germany; 6 https://ror.org/001w7jn25 grid.6363.0 0000 0001 2218 4662 Department of Gynecology with Breast Center, Charité – University Medicine Berlin, Berlin, Germany; 7 https://ror.org/05sxbyd35 grid.411778.c 0000 0001 2162 1728 Interdisciplinary Breast Center, University Medical Center Mannheim, Heidelberg University, Mannheim, Germany; 8 Breast Unit, Clinics Essen-Mitte, Essen, Germany; 9 Breast Center, Rotkreuz Clinics Munich, Munich, Germany; 10 University Clinics Leipzig, Women’s Clinic, Leipzig, Germany; 11 https://ror.org/02na8dn90 grid.410718.b 0000 0001 0262 7331 Breast Center, Department of Gynecology and Obstetrics, University Hospital of Essen, Essen, Germany; 12 Department of Oncology and Hematology, Niels Stensen-Kliniken, Georgsmarienhütte, Germany; 13 Praxis Dr. H. Forstbauer, C. Ziske, R. Reihs, E. Rodermann, A. Diel, Troisdorf, Germany; 14 University Clinics Tübingen, Women’s Clinic, Tuebingen, Germany; 15 https://ror.org/008xb1b94 grid.477277.6 0000 0004 4673 0615 . Elisabeth Hospital, Cologne, Germany; 16 https://ror.org/03cn8n632 grid.492783.3 Klinikum Mutterhaus Der Borromäerinnen, Trier, Germany; 17 Women’s Clinic and Breast Center, University Clinics Cologne, Cologne, Germany; 18 https://ror.org/0030f2a11 grid.411668.c 0000 0000 9935 6525 University Hospital Gynecology and Policlinic Rostock, Rostock, Germany; 19 Ev. Hospital Bergisch Gladbach, Bergisch Gladbach, Germany; 20 Johanniter Women’s Clinic Stendal, Stendal, Germany; 21 https://ror.org/05591te55 grid.5252.0 0000 0004 1936 973X Breast Center, Department of Gynecology and Obstetrics and CCCMunich, LMU University Hospital Munich, BZKF, Munich, Germany; 22 https://ror.org/01zgy1s35 grid.13648.38 0000 0001 2180 3484 Department of Gynecology, University Medical Center Hamburg, Hamburg, Germany; 23 https://ror.org/04839sh14 grid.473452.3 Institute of Biostatistics and Registry Research, Brandenburg Medical School Theodor Fontane, Neuruppin, Germany; 24 https://ror.org/01zgy1s35 grid.13648.38 0000 0001 2180 3484 Department of Medical Biometry and Epidemiology, University Medical Center Hamburg, Hamburg, Germany; 25 https://ror.org/02jet3w32 grid.411095.8 0000 0004 0477 2585 LMU University Hospital Munich, Munich, Germany; 26 https://ror.org/001w7jn25 grid.6363.0 0000 0001 2218 4662 Charité, Women’s Clinic, Berlin, Berlin, Germany; 27 https://ror.org/02na8dn90 grid.410718.b 0000 0001 0262 7331 Women’s Clinic, University Clinics Essen, Essen, Germany; 28 https://ror.org/054vayn55 grid.10403.36 0000000091771775 Translational Genomics and Targeted Therapies in Solid Tumors, August Pi I Sunyer Biomedical Research Institute (IDIBAPS), Barcelona, Spain; 29 Reveal Genomics, Barcelona, Spain; 30 https://ror.org/021018s57 grid.5841.8 0000 0004 1937 0247 Medicine Department, University of Barcelona, Barcelona, Spain; 31 Breast Cancer Unit, IOB-QuirónSalud, Barcelona, Spain; 32 https://ror.org/026zzn846 grid.4868.2 0000 0001 2171 1133 Queen Mary University of London, London, UK; 33 https://ror.org/01kc31v38 grid.428370.a 0000 0004 0409 2643 Exact Sciences, Redwood City, CA USA

## Abstract

**Background:**

New data show that not only HER2-overexpressing breast cancer (BC) tumors but also HER2-low tumors, classically considered as HER2-negative, respond to HER2-targeting antibody–drug-conjugates. Our objective was to analyze the prevalence of HER2-low BC in a pooled analysis of contemporary early BC trials and to evaluate its role as a prognostic factor in terms of survival in comparison to HER2-zero BC.

**Methods:**

We evaluated 5598 patients with locally HR + /HER2- BC from the screening cohort of WSG-ADAPT-HR + /HER2-, 2592 patients with HR + /HER2- or HR-/HER2- from the adjuvant WSG-PlanB trial, and 336 patients from the WSG-ADAPT-TN trial. Central HER2 testing was performed prospectively in WSG-ADAPT and retrospectively in WSG-PlanB. Following ASCO/CAP guidelines, HER2-low status was defined as immunohistochemistry (IHC) 1 + or 2 + and in situ hybridization (ISH)-negative, and HER2-zero was defined as IHC 0. Agreement between HER2 assessments was evaluated with Cohen’s kappa coefficient, and effects of HER2 status on pathological complete response (pCR) and on survival were analyzed with logistic regression and Cox proportional hazards models, respectively.

**Findings:**

In WSG-ADAPT-HR + /HER2-, 3198 (64.6%) tumors were HER2-low by the central and 3096 (55.6%) by the local histology (agreement for HER2-low status was 61.0%). In HR + /HER2- cases from WSG-PlanB, 601 tumors (28.7%) were HER2-low. In both cohorts, HER2-low status was significantly associated with higher *ERBB2* mRNA expression by Oncotype DX test in comparison to HER2-zero: mean 9.3 vs. 9.1 (p < .001) by local HER2 assessment in WSG-ADAPT and mean 9.2 vs. 8.8 (p < .001) in WSG-PlanB. Furthermore, patients with HER2-low tumors in WSG-ADAPT-HR + /HER2- significantly less often had a pCR compared to the HER2-zero tumors (p = .015). No significant difference was observed in (invasive and/or distant) disease-free survival (DFS) between centrally HER2-low and HER2-zero tumors in both HR + /HER2- cohorts (WSG-ADAPT-HR + /HER2- distant DFS: unadjusted HR = 1.06, 95%CI 0.83–1.36, similar results for local assessment; WSG-PlanB DFS: unadjusted HR = 1.28, 95%CI 0.91–1.82). In the HR-/HER2- WSG-PlanB cohort, centrally HER2-low tumors (10.5%) were associated with better DFS (unadjusted HR = 0.21, 95%CI 0.05–0.83), this association was not observed in the WSG-ADAPT-TN.

**Conclusion:**

The prevalence of HER2-low status varied between the analyzed trials. Our results show that survival does not differ between HER2-low and HER2-zero tumors in HR + /HER2- cohorts; however, HER2-low status appears to have an inconsistent impact on survival in TNBC. Therefore, our findings do not support the characterization of HER2-low status as a distinct BC subtype.

**Supplementary Information:**

The online version contains supplementary material available at 10.1186/s13058-025-01969-z.

## Introduction

Breast carcinoma is the most common malignant tumor disease in women [1–3]. A worldwide incidence of about 2.26 million cases in 2020, according to the global cancer burden, vividly demonstrates the extent of the disease and its global impact [4]. Different breast cancer subtypes are known, including the most common hormone receptor (HR)-positive, human epidermal growth factor receptor 2 (HER2)-negative type, to less frequent triple-negative breast cancer (HR-negative and HER2-negative) and HER2 − positive breast cancer [5].

HER2 is a prototype oncogene and an established therapeutic target in breast cancer (BC). Determination of the HER2 status in BC is a clinical routine that uses a combination of immunohistochemistry (IHC) to evaluate HER2 protein expression levels and in situ hybridization (ISH) to assess HER2 gene status [6, 7]. In 2007, the American Society of Clinical Oncology/College of American Pathologists (ASCO/CAP) released the first recommendations for HER2 testing for the distinction between HER2-negative and HER2-positive tumors [8]. The subsequent update in 2013 aimed at detecting and eliminating false negative cases by changing the cutoff for IHC score 3 + from 30 to 10% and changing the ISH HER2/CEP17 ratio ≥ 2.0 or HER2 absolute gene copy number ≥ 6.0 (previously HER2/CEP17 ratio ≥ 2.2 or HER2 average gene copy number ≥ 6.0)[9]. The 2018 update focused on the relevance of IHC score 0 versus 1 + by providing practical recommendations for testing and reporting [10]. The most recent 2023 update introduced the HER2-low category defined as IHC score 1 + or 2 + (ISH-negative)[11]. According to the current IHC algorithm, HER2-negative tumors are defined as tumors that are completely negative for HER2 (IHC score 0) as well as tumors with low (IHC score 1 +) or moderate HER2 expression (IHC score 2 + , ISH-negative). The minority of breast tumors are HER2-positive (15%) and show a HER2 overexpression with an IHC score 3 + or 2 + and ISH-positive. Until recently, IHC scores 0, 1 + , and 2 + with ISH-negative within the HER2-negative group have often been combined due to a lack of clinical relevance. However, a recent study (DESTINY-Breast04) showed that not only BC patients with HER2 overexpression but also patients with a low or moderate HER2 expression could benefit from antibody–drug conjugates like trastuzumab deruxtecan (T-DXd) [12]. Therefore, the question arises whether this HER2-low subgroup is a distinct entity different from HER2 IHC 0 BC.

The purpose of this study was to investigate whether patients with tumors with low HER2 expression (IHC 1 + or 2 + with ISH-negative) have a different clinical outcome compared to completely HER2-negative patients (HER2-zero, IHC 0). We also evaluated the agreement between the HER2 score derived from local and central pathologists and the association with the expression of the *ERBB2* gene on the mRNA level.

We used data from cohorts of patients with tumors originally classified as HER2- BC by local measurements, enrolled into three large clinical trials performed by the West Germany Study Group (WSG) in early BC: WSG-ADAPT-HR + /HER2- [13], WSG-ADAPT-TN [14], and adjuvant WSG-PlanB [15].

A standardized evaluation of HER2 expression in pre-therapeutic core biopsies has been performed prospectively in the WSG-ADAPT trials and retrospectively in the WSG-PlanB trial as a part of central pathology assessment. Endpoints included disease-free survival (DFS) and overall survival (OS). Additionally, we compared biologically relevant parameters, e.g., hormone receptor status, grade, tumor proliferation (Ki-67), and stromal tumor-infiltrating lymphocytes (sTILs) between the HER2-low and HER2-zero tumors.

## Methods

### Study design, clinical cohorts, and central pathology.

WSG-ADAPT-HR + /HER2- (NCT01779206) was a multi-center, randomized, open-label, phase II/III neoadjuvant trial in patients with primary unilateral invasive, locally hormone receptor (HR)-positive, and HER2-negative BC. Potential candidates for neoadjuvant chemotherapy (cT2-4 or cN + or G3 or Ki-67 ≥ 15%) were enrolled. Patients received 3 weeks of induction endocrine therapy (ET). Ki-67 was analyzed centrally in baseline diagnostic core biopsies and after the induction of ET, and patients with Ki-67_post-ET_ ≤ 10% were considered as ET-responders [16]. Patients with cN0-1 and either Recurrence Score (RS, Oncotype DX) ≤ 11 or RS between 12 and 25 with ET-response were included in the “endocrine sub-trial” and received no chemotherapy in either neoadjuvant or adjuvant phase. Patients with cN2-3 or RS > 25 or very-high-clinical-risk (cN2-3 OR (G3 AND baseline Ki-67 > 40% AND ≥ cT1c) or with ET-non-response (Ki-67_post-ET_ > 10%) entered the “chemotherapy sub-trial”. These patients were randomized with a 1:1 ratio to 8 weeks of intravenous sb-paclitaxel or nab-paclitaxel. Afterwards, patients received 4 cycles of epirubicin/cyclophosphamide (EC) q2w within a dose-dense chemotherapy schedule. Results for the primary endpoint, invasive DFS (iDFS), were published in 2022 [13].

WSG-PlanB (NCT01049425) was a multi-center, randomized, open-label, phase III adjuvant trial in patients with unilateral primary invasive BC with adequate surgical treatment and HER2-negative status, pT1-4c, known HR-status, pN + or pN0 with at least one risk factor (pT2 or greater, G2-3, high uPA/PAI-1, age < 35 years, or HR-negative status). Patients were randomized to 6 cycles of docetaxel plus cyclophosphamide q3w or to 4 cycles of EC q3w. Following the amendment, enrolled patients with HR + disease and RS < 12 received ET without chemotherapy. Results for the primary endpoint, DFS, were published in 2019 [17].

WSG-ADAPT-TN (NCT01815242) was a multi-center, randomized, open-label, phase II neoadjuvant trial in unilateral primary invasive, cT1c-cT4c or cN + centrally-confirmed triple-negative (TN) BC. Patients were randomized with a 1:1 ratio to nab-paclitaxel plus gemcitabine q3w or to nab-paclitaxel plus carboplatin q3w. Results for the primary endpoint, pathological complete response (pCR, defined as ypT0/is, ypN0, assessed after twelve weeks of treatment), were published in 2017 [14].

HER2 testing was conducted in each trial following the ASCO/CAP guidelines at that time: according to 2007 guidelines in WSG-PlanB [8] and according to 2007 and 2013 guidelines in WSG-ADAPT trials [8, 9]. In the WSG-ADAPT trials, central HER2 testing was performed prospectively before randomization. In the WSG-PlanB trial, central HER2 determination was performed retrospectively using tissue microarrays from surgical samples. In line with the German Gynecological Oncology Group guidelines (Arbeitsgemeinschaft Gynäkologische Onkologie, AGO, [18]), HER2-low status was defined for the purpose of this analysis as IHC 1 + or 2 + and ISH-negative, and HER2-zero was defined as IHC 0. HER2 IHC status was determined at diagnosis by a local and the first central assessment and after three weeks of treatment by the second central assessment. Additionally, the three IHC assessments were combined and defined as (i) HER2-negative if all three assessments were HER2-negative (IHC scores 0, 1 + , and 2 + with ISH-negative) or at least one assessment was HER2-negative while others were HER2-negative or had unclear HER2 status; (ii) HER2-positive if at least one assessment was HER2-positive; (iii) had unclear HER2 status if all three assessments were unclear; (iv) or were defined as HER2-low in the remaining cases.

Correlations between the different HER2 status evaluations were defined as follows: as no change if the first (diagnostic) and second (after three weeks of treatment) central assessments were identical; as a decrease if the first central assessment was HER2-low while the second central assessment was HER2-negative; as an increase if the first central assessment was HER2-negative while the second central assessment was HER2-low or HER2-positive or the first central assessment was HER2-low and the second central assessment was HER2-positive.

### Gene expression analysis

Expression of the *ERBB2* gene mRNA was evaluated in baseline tumor biopsies. Oncotype DX test (Exact Sciences, [19]) utilizing reverse transcriptase quantitative polymerase chain reaction (RT-qPCR) was used to analyze the expression of 21 genes in the WSG-ADAPT-HR + /HER2- trial and in the HR + cohort within the WSG-PlanB trial, as described previously [13, 20]. Customized nCounter 119-gene expression panels (NanoString Technologies Inc.) utilizing the multiplex nucleic acid hybridization, were used in the WSG-ADAPT-TN trial and in the HR- cohort within the WSG-PlanB trial [21, 22]. The customized 119-gene panel included targets for PAM50 intrinsic subtype predictor, Claudin-low subtype predictor, and VEGF/hypoxia signature (see https://www.ncbi.nlm.nih.gov/geo/query/acc.cgi?view=data&acc=GPL17071&id=42240&db=GeoDb_blob111), and nine immune-related genes (*PD1*, *PDL1*, *CD8*, *CD4*, *AR*, *FOXP3*, and three additional genes for PAM50). In both gene expression assays, the raw *ERBB2* gene expression data was normalized using the set of housekeeping genes: *ACTB*, *GAPDH*, *GUS*, *RPLPO*, and *TFRC* in the Oncotype DX assay and *ACTB*, *MRPL19*, *PSMC4*, *RPLP0*, and *SF3A1* in the nCounter assay. Additionally, *ERBB2* mRNA levels in a few patients in the HR + /HER2- cohort in the WSG-PlanB trial were analyzed by the PAM50 gene expression assay (NanoString Technologies Inc.).

### Statistical analysis

Patient’s and tumor’s characteristics were compared between HER2-negative and HER2-low using a Pearson’s Chi-square test, a Fisher’s exact test or a linear-by-linear test (categorical variables), and a t-test or Mann–Whitney-U test (continuous variables). Agreement between the different HER2 assessments was evaluated with a Cohen’s kappa coefficient, and the percentage agreement was calculated as a percentage of assessments with the same classification of HER2. Effects of HER2 on pCR occurrence were estimated with separate unadjusted and adjusted logistic regressions, while effects of HER2 on distant disease-free survival (dDFS), iDFS, DFS, and OS were estimated with separate unadjusted and adjusted Cox proportional hazards models. iDFS, dDFS, and OS were defined based on the STEEP criteria [23]. iDFS events (in WSG-ADAPT-TN) included local/regional invasive recurrence, distant recurrence, invasive ipsilateral recurrence, invasive contralateral BC, second primary invasive non-BC, death from any cause; dDFS events in (WSG-ADAPT-TN and WSG-ADAPT-HR + /HER2-) included distant recurrence, second primary invasive non-BC, death from any cause; and OS event (in WSG-PlanB and WSG-ADAPT-TN) was death from any cause. DFS in WSG-PlanB was defined with the following events: local, regional, or metastatic relapse, second primary cancer (with the exception of curatively treated non-melanoma skin cancer or in situ carcinoma of the cervix), and death from any cause. The adjusted effects of HER2 were obtained by including the patient’s and tumor’s characteristics that in univariable models had p-values < 0.10 and were significant in multivariable models. Additionally, interaction terms between HER2 and pCR and HER2 and treatment arm were tested in final multivariable models. Survival curves were presented with the Kaplan–Meier method and compared with a log-rank test. Furthermore, the prognostic effects of the HER2 dynamic on survival outcomes were evaluated using the same methodology. P-values ≤ 0.05 were considered statistically significant. All analyses were performed with STATA version 18.

## Results

A total of 5598 patients with HR + /HER2- BC (as per initial local assessment) from the screening cohort of WSG-ADAPT-HR + /HER2-, 2162 patients with HR + /HER2- and 430 patients with HR-/HER2- tumors from the adjuvant WSG-PlanB trial and 336 patients from the WSG-ADAPT-TN trial were evaluated. However, a small number of patients in each trial were missing the HER2 IHC data from local or central assessments.

### WSG-ADAPT-HR + /HER2- trial

**Fig. 1:**
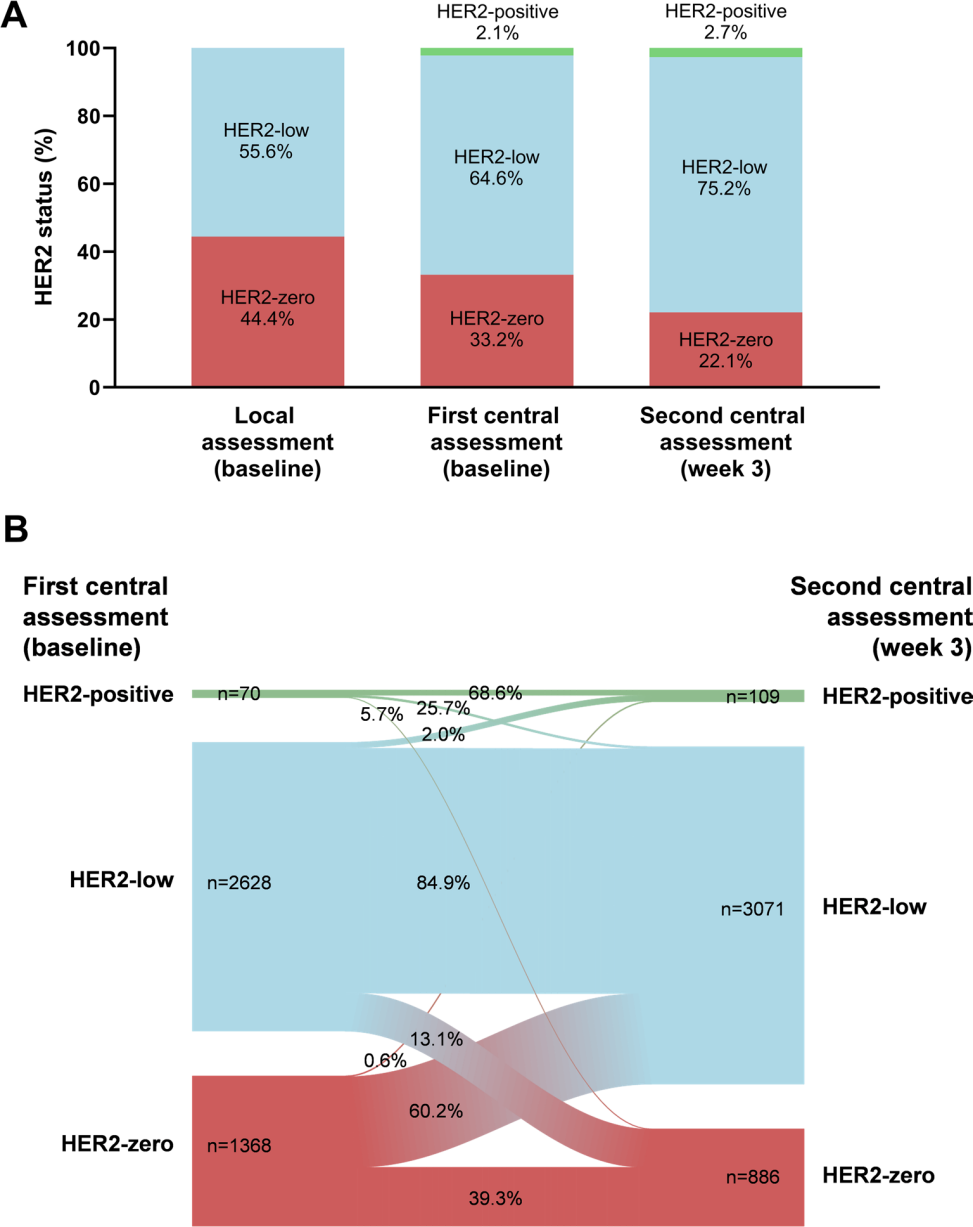
**A** HER2 assessment by local and the first and second central assessment and **B** changes in HER2 status between the first and the second central assessment in the WSG-ADAPT-HR + /HER2- trial

In the cohort of WSG-ADAPT-HR + /HER2-, a total of 55.6% (n = 3096 of 5571) and 64.6% (n = 3198 of 4948) of tumors were HER2-low (IHC 1 + or 2 + and ISH-negative) by local and the first central histological evaluation, respectively (Fig. 1A). 2.1% (n = 106 of 4948) of samples were identified as HER2-positive by the first central assessment and were thus excluded from further analysis. The agreement between the local and the first central evaluation for HER2-low and/or -zero was 61.0% (kappa 0.21, Table 1).

**Table 1:** Agreement between the local and the first central and the second central assessments in the WSG-ADAPT-HR + HER2- trial

Assessment		First central assessment (baseline)			
		HER2-zero	HER2-low	HER2-positive	Total
Local assessment (baseline)	HER2-zero	990 (60.3%)	1166 (36.7%)	24 (23.5%)	2180 (44.3%)
	HER2-low	651 (39.7%)	2013 (63.3%)	78 (76.5%)	2742 (55.7%)
	Total	1641 (33.3%)	3179 (64.6%)	102 (2.1%)	4922 (100%)
	Kappa			0.208	
Second central assessment (week 3)	HER2-zero	537 (39.3)	345 (13.1%)	4 (5.7%)	886 (21.8%)
	HER2-low	823 (60.1%)	2230 (84.9%)	18 (25.7%)	3071 (75.5%)
	HER2-positive	8 (0.6%)	53 (2.0%)	48 (68.6%)	109 (2.7%)
	Total	1368 (33.6%)	2628 (64.5%)	70 (1.7%)	4066 (100%)
	Kappa			0.298	

A second central assessment of HER2 status was carried out after a short (2–4 weeks) induction ET with tamoxifen in premenopausal and mostly aromatase inhibitors (AI) in postmenopausal women. At the second central assessment, 75.2% (n = 3262 of 4336) of tumors were classified as HER2-low (Fig. 1A). Thus, this assessment showed the highest frequency of HER2-low tumors in comparison to the baseline local and central assessment. The agreement between the first and the second central assessment was 69.2% (kappa 0.30, Table 1). 60.2% (n = 823 of 1368) of HER2-zero cases at baseline changed to HER2-low, but only 13.1% (n = 345 of 2628) of the cases changed from HER2-low to HER2-zero after the short ET, by central assessment (Fig. 1B).

Higher expression of HER2, with 35% of the tumors classified as IHC 2 + , was observed after ET in comparison to both baseline assessments that classified 24% of the tumors as IHC 2 + . The clinicopathological parameters for patients with local assessments of HER2 are shown in Table 2. The HER2-low cases showed slightly, but significantly higher *ERBB2* mRNA expression by Oncotype DX in comparison to HER2-zero cases (mean 9.3 vs. 9.1, p < 0.001, for the first local assessment, Table 2; mean 9.4 vs. 8.8, p < 0.001, for first central assessment; mean 9.3 vs. 8.9, p < 0.001, for second central assessment).

**Table 2:** Clinicopathological parameters in HR + cohorts and association with HER2-low status by local assessment in the WSG-ADAPT-HR + /HER2- trial and by first central assessment WSG-PlanB trial

Characteristic	WSG-ADAPT-HR + /HER2- trial				WSG-PlanB trial			
	HER2-zero, N = 2475	HER2-low, N = 3096	Overall, N = 5571*	p-value**	HER2-zero, N = 1468	HER2-low, N = 601	Overall, N = 2069***	p-value****
Age (years)								
Mean (SD)	55.3 (10.3)	54.6 (10.4)	54.9 (10.4)	.038	55.8 (10.1)	55.4 (9.7)	55.7 (10.0)	.432
Median (min, max)	54 (23, 84)	54 (20, 85)	54 (20, 85)		55.5 (27, 77)	55 (25, 76)	55 (25, 77)	
Estrogen receptor (%)								
Mean (SD)	90.4 (17.3)	90.7 (16.8)	90.6 (17.1)	.557	90.6 (21.1)	92.4 (17.8)	91.1 (20.2)	.042
Median (min, max)	100 (0, 100)	100 (0, 100)	100 (0, 100)		100 (0, 100)	100 (0, 100)	100 (0, 100)	
Missing	295 (11.9%)	344 (11.1%)	639 (11.5%)		7 (0.5%)	0 (0.0%)	7 (0.3%)	
Progesterone receptor (%)								
Mean (SD)	64.4 (36.6)	62.2 (36.7)	63.2 (36.6)	.031	62.7 (41.7)	62.9 (40.4)	62.8 (41.3)	.948
Median (min, max)	80 (0, 100)	80 (0, 100)	80 (0, 100)		80 (0, 100)	80 (0, 100)	80 (0, 100)	
Missing	288 (11.6%)	348 (11.2%)	636 (11.4%)		11 (0.8)	3 (0.5%)	14 (0.7%)	
Ki-67, baseline (%)								
Mean (SD)	22.5 (14.9)	22.2 (14.3)	22.3 (14.6)	.436	15.8 (11.0)	16.9 (11.1)	16.1 (11.0)	.057
Median (min, max)	20 (0, 95)	20 (0, 100)	20 (0, 100)		15 (1, 95)	15 (1, 80)	15 (1, 95)	
Missing	413 (16.7%)	452 (14.6%)	865 (15.5%)		83 (5.7%)	18 (3.0%)	101 (4.9%)	
Ki-67, week 3 (%)								
Mean (SD)	12.5 (13.2)	12.4 (12.2)	12.4 (12.6)	.713	NA	NA	NA	NA
Median (min, max)	10 (0, 95)	10 (0, 90)	10 (0, 95)		NA	NA	NA	
Missing	577 (23.3%)	710 (22.9%)	1287 (23.1%)		NA	NA	NA	
RS								
Mean (SD)	19.2 (10.9)	20.3 (10.8)	19.8 (10.9)	< .001	18.9 (10.1)	20.1 (10.1)	19.2 (10.1)	.016
Median (min, max)	17 (0, 75)	18 (0, 76)	18 (0, 76)		18 (0, 69)	18 (0, 99)	18 (0, 99)	
Missing	282 (11.4%)	324 (10.5%)	606 (10.9%)		47 (3.2%)	13 (2.2%)	60 (2.9%)	
*ERBB2* mRNA by Oncotype DX								
Mean (SD)	9.1 (0.7)	9.3 (0.7)	9.2 (0.7)	< .001	8.8 (0.7)	9.2 (0.7)	8.9 (0.7)	< .001
Median (min, max)	9.1 (6.9, 11.9)	9.4 (7.6, 12.9)	9.2 (6.9, 12.9)		8.9 (5.5, 12.7)	9.2 (6.9, 11.7)	9 (5.5, 12.7)	
Missing	611 (24.7%)	793 (25.6%)	1404 (25.2%)		62 (4.2%)	15 (2.5%)	77 (3.7%)	
*ERBB2* mRNA by PAM50*****								
Mean (SD)	NA	NA	NA	NA	-1.8 (0.8)	-0.3 (1.3)	-1.4 (1.1)	.005
Median (min, max)	NA	NA	NA		-1.9 (-2.8, 0.8)	-0.5 (-1.6, 2.0)	-1.7 (-2.8, 2.0)	
Missing	NA	NA	NA		1448 (98.6%)	595 (99.0%)	2043 (98.7%)	
Histology Type								
NST	1749 (80.6%)	2304 (84.1%)	4053 (82.5%)	.014	1226 (83.7%)	519 (86.4%)	1745 (84.5%)	.304
ILC	367 (16.9%)	383 (14.0%)	750 (15.3%)		201 (13.7%)	70 (11.7%)	271 (13.1%)	
Mucinous	31 (1.4%)	30 (1.1%)	61 (1.2%)		0 (0.0%)	0 (0.0%)	0 (0.0%)	
Other	24 (1.1%)	23 (0.8%)	47 (1.0%)		38 (2.6%)	12 (2.0%)	50 (2.4%)	
Missing	304 (12.3%)	356 (11.5%)	660 (11.9%)		3 (0.2%)	0 (0.0%)	3 (0.1%)	
Histological grade								
0–1	146 (8.1%)	171 (7.6%)	317 (7.8%)	.687	83 (5.7%)	33 (5.5%)	116 (5.6%)	.063
2	979 (54.2%)	1260 (55.9%)	2239 (55.2%)		919 (62.6%)	348 (57.9%)	1267 (61.2%)	
3	682 (37.7%)	822 (36.5%)	1504 (37.0%)		466 (31.7%)	220 (36.6%)	686 (33.2%)	
Missing	668 (27.0%)	843 (27.2%)	1511 (27.1%)		0 (0.0%)	0 (0.0%)	0 (0.0%)	
Tumor stage******								
0/is	26 (1.3%)	10 (0.4%)	36 (0.8%)	.984	0 (0.0%)	0 (0.0%)	0 (0.0%)	.161
1	1081 (52.5%)	1366 (53.7%)	2447 (53.2%)		785 (53.6%)	339 (56.6%)	1124 (54.5%)	
2	856 (41.6%)	1059 (41.6%)	1915 (41.6%)		611 (41.7%)	238 (39.7%)	849 (41.2%)	
3–4	97 (4.7%)	108 (4.3%)	205 (4.5%)		68 (4.6%)	22 (3.7%)	90 (4.4%)	
Missing	415 (16.8%)	553 (17.9%)	968 (17.4%)		4 (0.3%)	2 (0.3%)	6 (0.3%)	
Nodal stage******								
0	1674 (67.6%)	2060 (66.6%)	3734 (67.0%)	.773	817 (55.7%)	354 (58.9%)	1171 (56.6%)	.044
1	612 (24.7%)	817 (26.4%)	1429 (25.7%)		539 (36.7%)	218 (36.3%)	757 (36.6%)	
2–3	189 (7.6%)	218 (7.0%)	407 (7.3%)		112 (7.6%)	29 (4.8%)	141 (6.8%)	
Missing	0 (0.0%)	1 (0.0%)	1 (0.0%)		0 (0.0%)	0 (0.0%)	0 (0.0%)	
pCR (ypT0/is, ypN0), neoadjuvant treated patients only								
No	299 (79.5%)	417 (85.8%)	716 (83.1%)	.015	NA	NA	NA	NA
Yes	77 (20.5%)	69 (14.2%)	146 (16.9%)		NA	NA	NA	
Missing	2099 (84.8%)	2610 (84.3%)	4709 (84.5%)		NA	NA	NA	

Percentages for categories other than missing were obtained among non-missing observations and they sum up to 100%

^*^27 patients (0.5%) were classified as having unclear HER2 status and were excluded from the analyses

^**^P-values were obtained with a t-test (continuous variables) and a Pearson’s Chi-square test or a linear-by-linear test (categorical variables)

^***^28 patients (1.3%) were classified as HER2-positive, and 65 (3.0%) patients were classified as having unclear HER2 status and were excluded from the analyses

^****^P-values were obtained with a t-test or Mann–Whitney-U test (continuous variables) and a Pearson’s Chi-square test or a linear-by-linear test (categorical variables). Missing categories were excluded from statistical testing

^*****^ERBB2 mRNA levels were analyzed by PAM50 in locally or centrally HR- cases in the WSG-PlanB trial

^******^Denotes composite tumor or nodal stage for WSG-ADAPT-HR + /HER2- trial (clinical stage in patients randomized to neoadjuvant treatment, pathological stage in patients after primary surgery); pathological tumor or nodal stage in WSG-PlanB trial

NST, non-special type carcinoma; ILC, invasive lobular carcinoma

A combination of all three HER2 assessments classified 83.5% (n = 4675 of 5598) of the tumors as HER2-low. Importantly, HER2-positive status was found in an additional 66 (1.2%) surgical samples.

Ki-67 levels at baseline or at week 3 did not differ between HER2-zero and HER2-low cases.

Patients with HER2-low tumors treated by neoadjuvant chemotherapy (mostly dose-dense paclitaxel-EC or 8 × nab-paclitaxel, q1w—4xEC, q2w) had significantly less often a pCR compared to the HER2-zero patients (14.2% vs. 20.5%; p = 0.015, Table 2), and there was no interaction with the therapy arm.

However, after a median of 61 months follow-up, there was no significant difference in dDFS between HER2-low and HER2-zero tumors by neither local (unadjusted HR = 0.95, 95%CI 0.76–1.18) and central first (unadjusted HR = 1.06, 95%CI 0.83–1.36) nor central second assessment (unadjusted HR = 1.04, 95%CI 0.77–1.41, Supplementary Fig. 1A-C). These results were also true if analyzed separately by ET and chemoendocrine therapy. Moreover, the dynamics of HER2 expression (assessed centrally at baseline and at week 3 of ET) were not associated with survival outcomes (Supplementary Fig. 1D).

### HR + /HER2- cohort in the WSG-PlanB trial

In the HR + /HER2- cohort of WSG-PlanB, 28.7% of tumors (n = 601 of 2097) were HER2-low by first central assessment (see Table 2 for the clinicopathological characteristics of patients). 15.6% (n = 94 of 601) of the HER2-low cases were classified as IHC 2 + , and the HER2-low tumors had significantly higher *ERBB2* mRNA expression by Oncotype DX in comparison to HER2-zero tumors (mean 9.2 vs. 8.8, p < 0.001, Table 2). However, no significant difference was observed in OS and DFS between HER2-low and HER2-zero tumors (OS: unadjusted HR = 1.21, 95%CI 0.73–2.02; DFS: unadjusted HR = 1.28, 95%CI 0.91–1.82, Supplementary Fig. 2A, B).

Moreover, no interaction with the efficacy of anthracycline-containing or anthracycline-free treatment was observed neither with immunohistochemical- (p = 0.776) nor with RT-qPCR-based HER2 expression (p = 0.918). However, significantly worse DFS was observed for higher HER2 expression by RT-qPCR in 317 patients with up to 3 positive lymph nodes and RS 0–11 treated by ET alone (unadjusted HR for continuous levels = 2.35, 95%CI 1.10–5.00).

### HR-/HER2- cohort in the WSG-PlanB trial

In the HR-/HER2- cohort of WSG-PlanB, only 10.5% (n = 45 of 430) of cases were HER2-low and 1.4% (n = 6 of 430) were HER2-positive by central assessment. Of all the baseline characteristics studied, only *ERBB2* mRNA expression by nCounter assay was found to be significantly related to HER2 status; *ERBB2* mRNA expression was higher in HER2-low than in HER2-zero tumors (mean -1.0 vs. -2.0; p < 0.001). Patients with HER2-low tumors had significantly better DFS (unadjusted HR = 0.21, 95%CI 0.05–0.83) but not OS (unadjusted HR = 0.38, 95%CI 0.09–1.59) in comparison to HER2-zero cases (Supplementary Fig. 3A, B).

### WSG-ADAPT-TN trial

**Fig. 2:**
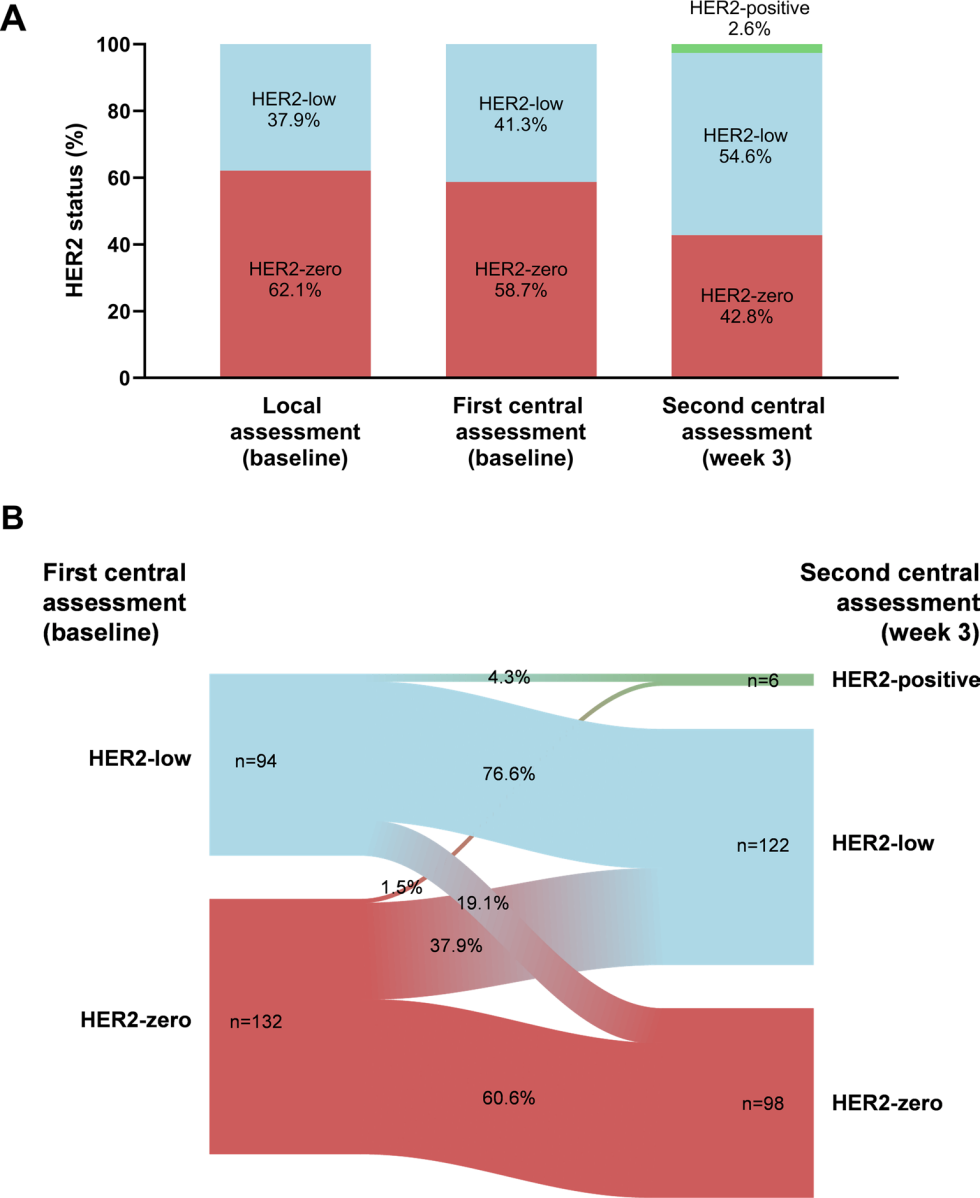
**A** HER2 assessment by local and the first and second central assessment and **B** changes in HER2 status between the first and the second central assessment in the WSG-ADAPT-TN trial

In the HR-/HER2- cohort of the WSG-ADAPT-TN trial, HER2-low status at baseline was found in 37.9% (n = 127 of 335) of cases by local assessment and 41.3% (n = 137 of 332) by the first central assessment with overall agreement of 65% (kappa 0.28, Fig. 2, Table 3). Moreover, 2.6% (n = 6 of 229) of tumors turned out to be HER2-positive, and 54.6% (n = 125 of 229) were found to be HER2-low by the second biopsy after 3 weeks of neoadjuvant chemotherapy. The agreement between central assessment at the first and second samples was 67% (kappa 0.37, Table 3). In general, 67.6% (n = 227 of 336) of tumors turned out to be HER2-low by at least one of the three assessments.

**Table 3:** Agreement between the local and the first central and the second central assessments in the WSG-ADAPT-TN trial

Assessment	First central assessment (baseline)		
	HER2-zero	HER2-low	Total
Local assessment (baseline)			
HER2-zero	142 (73.2%)	63 (46.0%)	205 (61.9%)
HER2-low	52 (26.8%)	74 (54.0%)	126 (38.1%)
Total	194 (58.6%)	137 (41.4%)	331 (100.0%)
Kappa		0.275	
Second central assessment (week 3)			
HER2-zero	80 (60.6%)	18 (19.2%)	98 (43.4%)
HER2-low	50 (37.9%)	72 (76.6%)	122 (54.0%)
HER2-positive	2 (1.5%)	4 (4.3%)	6 (2.7%)
Total	132 (58.4%)	94 (41.6%)	226 (100.0%)
Kappa		0.373	

Only the first central assessment of HER2 was significantly associated with *ERBB2* mRNA levels by nCounter assay with HER2-low cases having a higher *ERBB2* mRNA expression in comparison to HER2-zero (mean 0.3 vs. -0.2, p = 0.001, Table 4). Regarding association with biological patterns, we found significantly lower Ki-67 levels in HER2-low than HER2-zero cases by all three assessments (see Table 5 for the clinicopathological characteristics of patients). Locally assessed HER2-low status was associated with lower odds of pCR occurrence; however, only when the effect was not adjusted for any patient’s or tumor’s characteristics (unadjusted OR = 0.62, 95%CI 0.38–1.00; adjusted OR = 0.79, 95%CI 0.45–1.40). This effect was not observed with central assessments.

**Table 4:** Comparison of *ERBB2* mRNA expression between HER2-zero and HER2-low tumors by the local and central assessments in the WSG-ADAPT-TN trial

HER2 status	*ERBB2* mRNA expression by nCounter assay			
	Mean (SD)	Median (min, max)	Missing	p-value
Local assessment (baseline)				
HER2-zero	− 0.08 (0.94)	− 0.46 (− 0.82, 5.97)	17 of 208 (8%)	.083
HER2-low	0.13 (1.09)	− 0.26 (− 0.82, 3.78)	13 of 127 (10%)	
First central assessment (baseline)				
HER2-zero	− 0.17 (0.77)	− 0.46 (− 0.82, 4.00)	16 of 195 (8%)	.001
HER2-low	0.26 (1.22)	− 0.23 (− 0.82, 5.97)	13 of 137 (9%)	
Second central assessment (week 3)				
HER2-zero	− 0.05 (0.93)	− 0.39 (− 0.82, 4.00)	4 of 98 (4%)	.134
HER2-low	0.17 (1.18)	− 0.26 (− 0.81, 5.97)	9 of 125 (7%)	
Any assessment				
HER2-zero	− 0.20 (0.76)	− 0.46 (− 0.82, 4.00)	4 of 103 (4%)	.006
HER2-low	0.09 (1.08)	− 0.31 (− 0.82, 5.97)	26 of 227 (11%)

**Table 5:** Clinicopathological parameters and association with HER2-low status by local and by the first and the second central assessment in the WSG-ADAPT-TN trial

Baseline characteristics	Local assessment (baseline)*			First central assessment (baseline)**			Second central assessment (week 3)***		
	HER2-zero, N = 208	HER2-low, N = 127	p-value****	HER2-zero, N = 195	HER2-low, N = 137	p-value ****	HER2-zero, N = 98	HER2-low, N = 125	p-value ****
Age (years)									
Mean (SD)	50.8 (11.7)	52.2 (11.7)	.319	50.6 (11.6)	52.5 (11.8)	.145	51.6 (12.0)	50.5 (10.8)	.462
Median (min, max)	50 (26, 76)	53 (26, 75)		50 (27, 76)	53 (26, 75)		50 (29, 76)	50 (26, 75)	
Ki-67, baseline (%)									
Mean (SD)	69.9 (20.3)	60.3 (23.2)	< .001	69.6 (20.4)	62.2 (23.0)	.003	70.4 (19.7)	61.0 (24.7)	.002
Median (min, max)	75 (15, 100)	65 (10, 95)		75 (15, 95)	65 (10, 100)		75 (20, 95)	65 (10, 95)	
Missing	7 (3.4%)	5 (3.9%)		6 (3.1%)	4 (2.9%)		0 (0.0%)	6 (4.8%)	
sTILs, baseline									
Mean (SD)	29.7 (23.7)	28.7 (25.1)	.727	30.7 (24.8)	28.2 (23.8)	.363	30.6 (25.5)	28.1 (23.7)	.452
Median (min, max)	25 (0, 90)	20 (0, 90)		25 (0, 90)	20 (0, 90)		20 (0, 90)	20 (0, 90)	
Missing	7 (3.4%)	6 (4.7%)		5 (2.6%)	7 (5.1%)		1 (1.0%)	4 (3.2%)	
Treatment arm									
Gemcitabine	111 (53.4%)	70 (55.1%)	.755	113 (58.0%)	69 (50.4%)	.172	62 (63.3%)	71 (56.8%)	.329
Carboplatin	97 (46.6%)	57 (44.9%)		82 (42.0%)	68 (49.6%)		36 (36.7%)	54 (43.2%)	
Histological subtype									
Invasive ductal carcinoma	202 (98.5%)	124 (97.6%)	.678	191 (98.5%)	134 (97.8%)	.695	95 (96.9%)	121 (98.4%)	.657
Other	3 (1.5%)	3 (2.4%)		3 (1.5%)	3 (2.2%)		3 (3.1%)	2 (1.6%)	
Missing	3 (1.4%)	0 (0.0%)		1 (0.5%)	0 (0.0%)		0 (0.0%)	2 (1.6%)	
Histological grading									
2	11 (5.3%)	11 (8.7%)	.236	10 (5.1%)	11 (8.0%)	.285	3 (3.1%)	13 (10.5%)	.038
3	195 (94.7%)	116 (91.3%)		185 (94.9%)	126 (92.0%)		95 (96.9%)	111 (89.5%)	
Missing	2 (1.0%)	0 (0.0%)		0 (0.0%)	0 (0.0%)		0 (0.0%)	1 (0.8%)	
Clinical tumor stage									
1	76 (36.5%)	48 (37.8%)	.817	77 (39.5%)	47 (34.3%)	.337	38 (38.8%)	38 (30.4%)	.190
2–4	132 (63.5%)	79 (62.2%)		118 (60.5%)	90 (65.7%)		60 (61.2%)	87 (69.6%)	
Clinical nodal status									
0	156 (75.0%)	92 (72.4%)	.604	146 (74.9%)	99 (72.3%)	.595	69 (70.4%)	90 (72.0%)	.794
1–3	52 (25.0%)	35 (27.6%)		49 (25.1%)	38 (27.7%)		29 (29.6%)	35 (28.0%)	
pCR (ypT0/is, ypN0)									
No	120 (59.7%)	86 (70.5%)	.05	118 (63.1%)	85 (63.9%)	.882	69 (71.1%)	91 (73.4%)	.71
Yes	81 (40.3%)	36 (29.5%)		69 (36.9%)	48 (36.1%)		28 (28.9%)	33 (26.6%)	
Missing	7 (3.4%)	5 (3.9%)		8 (4.1%)	4 (2.9%)		1 (1.0%)	1 (0.8%)	

Percentages for categories other than missing were obtained among non-missing observations and they sum up to 100%

^*^1 patient (0%) was classified as having unclear HER2 status and was excluded from the analyses

^**^4 patients (1.2%) were classified as having unclear HER2 status and were excluded from the analyses

^***^6 patients (1.8%) were classified as HER2-positive and 107 (31.9%) patients were classified as having unclear HER2 status and were excluded from the analyses

^****^P-values were obtained with a t-test (continuous variables) and a Pearson’s Chi-square test or a Fisher’s exact test (categorical variables). Missing categories were excluded from statistical testing

After 60 months of median follow-up, neither iDFS nor OS were significantly associated with HER2 status by first local and second central assessment (Supplementary Fig. 4), as well as no significant interaction between study arm and pCR status was found. However, in comparison to HER2-zero cases, HER2-low cases assessed by second central assessment had a significantly lower risk for iDFS event up to first two years of follow-up (at 1 year: adjusted HR = 0.28, 95%CI 0.14–0.58; at 2 years adjusted: HR = 0.52, 95%CI 0.31–0.89) but not after longer follow-up, when the effect was adjusted for pCR, clinical node status and sTILs measured at baseline (Supplementary Fig. 4B). This effect significantly differed between carboplatin-free and carboplatin-containing arms up to 1-year follow-up (interaction test at 1-year follow-up: p = 0.023). It was highly pronounced (weaker regarding pCR) in the carboplatin-free arm (adjusted HR = 0.20, 95%CI 0.08–0.54), but not in the carboplatin-containing arm (adjusted HR = 0.85, 95%CI 0.40–1.82, Supplementary Fig. 5A and B). Similarly, a significant effect on dDFS of HER2 assessed by the second central assessment was observed (adjusted HR = 0.47, 95%CI 0.27–0.80) irrespective of pCR (none of HER2-low pCR cases experienced dDFS event, Supplementary Fig. 5C).

**Fig. 3:**
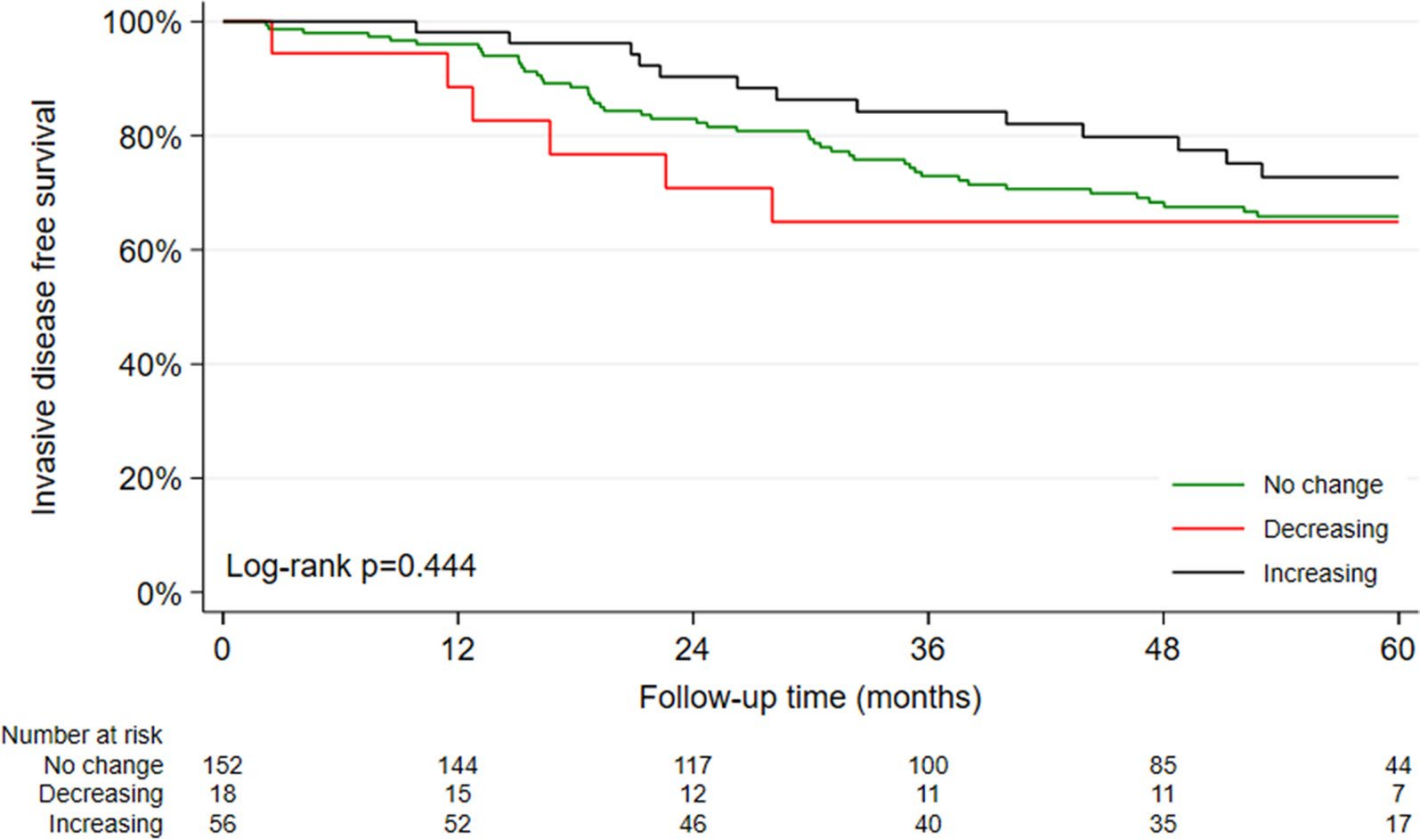
iDFS according to changes in HER2 status between the first and the second central IHC assessment in patients from the WSG-ADAPT-TN trial

Furthermore, the dynamics of HER2 expression had no significant impact on iDFS, dDFS, or OS. However, an increase in HER2 expression during therapy seemed to be associated with better survival outcomes (Fig. 3).

## Discussion

For the first time, we have reported results on HER2-low status from three large prospective contemporary trials in HER2-negative early BC according to both central and local assessments and its relationship to *ERBB2* mRNA expression in different genomic signatures.

The first important finding from our study is a clinically meaningful difference in HER2-low status between the older WSG-PlanB trial, which used tissue microarrays performed on surgical samples for central assessment of HER2, and the more current WSG-ADAPT trial, which determined HER2 on complete core biopsies as first assessment. Therefore, different tissue formats used for HER2 assessment in WSG-ADAPT and in the WSG-PlanB trial may well explain the observed differences in HER2-low frequency.

Although a positive association between HER2-low status and *ERBB2* mRNA expression was found in all four analyzed data sets, we have seen a significantly lower HER2-low prevalence in the WSG-PlanB trial (28.7% and 10.5% in HR + and HR- samples, respectively) vs. the WSG-ADAPT trial (64.6% and 41.3% in HR + and HR- subsets, respectively). This is despite the fact that the same central lab evaluated the HER2 expression and that HER2 levels by IHC positively correlated with HER2 expression by RT-qPCR from the Oncotype DX test. The HER2-low rate in the WSG-ADAPT, but not in the WSG-PlanB trial, appears comparable to those published by Denkert et al. in the large analysis by the German Breast Group (64% and 36% in the HR + and HR- subsets, respectively) [24] and comparable to the meta-analysis published by Xia et al. (66.5% and 33.5% in the HR + and HR-group)[25]. Similarly, Tarantino et al. reported HER2-low rates of 40% in HR- tumors and 62% in tumors with ER expression > 95% [26]. Recently, National Cancer Data Base (NCDB) analysis demonstrated a 70% HER2-low rate in HR + /HER2- cases [27].

Interestingly, we have observed a strong increase in HER2 expression with up to 75.2% of HER2-low cases after induction ET with AI or tamoxifen in the WSG-ADAPT-HR + /HER2- trial. These results are in line with the single-center phase II trial published by Chaudhary et al., who reported an increase in HER2 expression in 49% of cases after neoadjuvant ET [28]. A similar effect was observed in the WSG-ADAPT-TN cohort, with 37.9% of HER2-zero tumors by central assessment becoming HER2-low after 3 weeks of neoadjuvant chemotherapy. A significant dynamics of HER2 expression and a very high rate of HER2-low status, if several biopsies/analyses were repeated, has also recently been found by Bar et al. [29]. Several pathomechanisms could drive an increased HER2 expression during the ET. For instance, HER2 seems to activate nuclear factor kappa B (NF-κB), which in turn contributes to resistance against ET [30]. Therefore, it is possible that increased HER2 levels during induction ET may result from the expansion of HER2-expressing tumor cells with activated NF-κB signaling. Given the very promising activity of T-DXd and trastuzumab duocarmazine in apparently HER2-negative disease [31, 32], it would interesting to see whether different patterns of transition between HER2-zero and HER2-low status affect the efficacy of these antibody–drug conjugates. Still, several mechanisms of resistance to T-DXd, including inhibition of ferroptosis, have been described [33] and could limit the activity of antibody–drug conjugates in HER2-low disease.

However, the overall concordance for HER2-low status between local and central assessments was relatively low, despite a similar prevalence of HER2-low status in both assessments and a similar correlation with *ERBB2* expression by RT-qPCR. A lack of awareness regarding the importance of distinguishing between the HER2-zero and HER2-low subgroups among the HER2-negative cases (at the time when the trials were performed) likely contributed to the observed discordance between local and central results. This problem could be further compounded by the heterogeneous patterns of HER2 expression [34]. Overall, this stresses the need for standardization of HER2 testing in HER2-negative tumors in order to increase the concordance between the assessments. Though criteria for HER2 scoring have been clearly reported by current ASCO/CAP guidelines, it is still debated whether such methods (i.e., IHC and ISH assay) are appropriate for the detection of low levels of HER2 expression. Novel quantitative assays are currently under development and investigation, with the aim of improving the accuracy of HER2 testing [35, 36]. Alternatively, adequate training of pathologists has been recently shown to improve the diagnostic accuracy for identifying HER2-low cases [37]. A recent retrospective study by Viale et al. has shown that about 30% of historically HER2-zero cases would be rescored as HER2-low by a second assessment [38]. Another recent study provides excellent concordance only in tumors with HER2 IHC 3 + , but not in cases with lower expression [39].

Type of samples (core biopsy vs. surgery sample) may also play a role in observed different positivity rates, however our data on the second sample in the WSG-ADAPT trial (mostly surgery samples after ET) and also other studies do not support lower HER2 expression on the surgery samples [40] due to methodological issues (e.g. fixation).

Regarding the pCR rate, we have observed lower response rates in HER2-low vs HER2-zero cases in HR + /HER2- disease, in line with other publications [24, 26, 36] as well as a recently published meta-analysis [41]. However, this effect was pronounced only in tumors with locally assessed HER2-low status, as well as in samples after induction ET. Interestingly, another meta-analysis demonstrated lower pCR rates in HER2-low tumors regardless of the hormone receptor status [25]. A highly preselected patient population in the HR + /HER2- cohort in the WSG-ADAPT-HR + /HER2- trial makes any kind of cross-trial comparisons difficult. Impact on pCR was inconsistent in HR-/HER2- disease (found only in the HER2-low by local assessment but not confirmed by central assessment), which was in line with comparable pCR rates in TNBC published by Denkert et al. and Viale et al. [24, 38].

Moreover, we did not find a significant association between HER2-low status and iDFS in both WSG-ADAPT-HR + /HER2- and WSG-PlanB HR + /HER2- cohorts by any of the assessments, which is in line with some other studies [24, 36, 42–44]. In both presented analyses from the NCDB, a better outcome in the HER2-low group was pronounced in patients treated by chemotherapy and/or in those with RS > 25 [27], or in patients with higher stages of disease [45]. Therefore, patient preselection in both WSG trials may play some role. However, a meta-analysis published currently by Molinelli et al. shows significantly better iDFS and OS in both HR + and HR- cohorts in favor of HER2-low group [41].

In our HR-/HER2- cohorts, a significantly better DFS for HER2-low cases was found in the WSG-PlanB trial (with only 10% HER2-low rate), in line with previously mentioned and other studies [24, 25], but not in the overall analysis of the WSG-ADAPT-TN cohort with clearly higher HER2-low rates. However, a positive association between HER2-low status and pCR, particularly within the carboplatin-free arm, was found within the exploratory time-dependent multivariable analysis, including pCR. Importantly, as HER2-low status appeared highly dynamic between pretreatment and on-treatment biopsy (with up to 68% of all tumors found as HER2-low by one of three assessments), no significant association between change in HER2-low status and iDFS was found, but increasing HER2 expression was associated with a non-significant trend towards a better outcome compared to decreasing HER2 levels. To summarize, there was no consistent correlation between HER2-low status and survival outcomes in our data set. These partially divergent results can be explained by different chemotherapy regimens used in the different cohorts as well as by different study designs. However, large differences in HER-low rates in different studies (from 10% up to 100%) as well as dynamics of HER2 expression during treatment indicate that HER2 levels can serve as a marker for different biological processes in the tumor, even in TNBC, rather than as a biological driver of these processes.

Our data, along with others, suggest that ongoing research is essential to improve the accuracy of identifying HER-low cases. Efforts should concentrate on further enhancing reproducibility, reducing interobserver variability, and investigating the role of HER2 expression heterogeneity. Moreover, a better molecular characterization of HER2-low tumors is needed to identify patients most likely to respond to novel antibody–drug conjugates and explore the mechanism of resistance.

Our study has several limitations. For instance, the difference in percentages of HER2-low tumors between the WSG-PlanB and WSG-ADAPT trials could be due to the use of tissue microarrays on surgical specimens in the former and core biopsies in the latter trial. Dissimilar HER2-low frequency between the local and central assessments could result from a former lack of awareness regarding the importance of classifying HER2-negative tumors into HER2-zero and HER2-low cases. Moreover, different methods were used for the analysis of *ERBB2* gene expression levels (Oncotype DX test in HR + cohorts and customized nCounter panel and PAM50 assay in HR- cohorts), which precludes the comparison between the trials.

## Conclusions

In conclusion, our analysis of 8526 patients, as the largest data set reported so far to our best knowledge from randomized trials with consistent central and genomic assessment and survival results, does not support the concept that HER2-low is a distinct BC subtype. HER2-low BC differs from HER2-zero BC in terms of slightly higher ERBB2/HER2 mRNA expression; however, it does not define a distinct subtype. Moreover, very recently presented results from the DESTINY-06 trial show high efficacy of T-DXd in patients with both HER2-low and even HER2-ultralow expression in metastatic HR + /HER2- disease compared to first-line chemotherapeutic treatment. This indicates that HER2 expression is a very dynamic and heterogeneous factor, which could possibly explain the efficacy of antibody–drug conjugates also in apparently HER2-negative tumors.

## Supplementary Information

Supplementary Figure 1

Supplementary Figure 2

Supplementary Figure 3

Supplementary Figure 4

Supplementary Figure 5

## Data Availability

No datasets were generated or analysed during the current study.

## Abbreviations

AI: Aromatase inhibitors
ASCO/CAP: American Society of Clinical Oncology/College of American Pathologists
BC: Breast cancer
DFS: Disease-free survival
dDFS: Distant disease-free survival
EC: Epirubicin/cyclophosphamide
ET: Endocrine therapy
HER2: Human epidermal growth factor receptor 2
HR: Hormone receptor
iDFS: Invasive disease-free survival
IHC: Immunohistochemistry
ILC: Invasive lobular carcinoma
ISH: In situ hybridization
NCDB: National Cancer Data Base
NST: Non-special type carcinoma
OS: Overall survival
pCR: Pathological complete response
RS: Recurrence Score
sTILs: Stromal tumor-infiltrating lymphocytes
T-DXd: Trastuzumab deruxtecan
TNBC: Triple-negative breast cancer
WSG: West Germany Study Group
