# Supplementary material for: HER2-low status as a distinct breast cancer subtype: myth or truth? Analysis of the WSG trials WSG-ADAPT-HR+/HER2-, WSG-PlanB, and WSG-ADAPT-TN
Source: Breast Cancer Res. 2025 Feb 14;27:22. doi: 10.1186/s13058-025-01969-z (PMC11827153; doi:10.1186/s13058-025-01969-z)
Supplement: Supplementary file 1 — Supplementary Figure 1 [file 13058_2025_1969_MOESM1_ESM.docx]

Supplementary Figure 1. Comparison of dDFS between HER2-low and HER2-zero tumors by local (A), first (B), and second (C) central assessments, and according to changes in HER2 status between the first and the second central IHC assessment (D) in patients from the WSG-ADAPT-HR+/HER2- trial.
