# Supplementary material for: HER2-low status as a distinct breast cancer subtype: myth or truth? Analysis of the WSG trials WSG-ADAPT-HR+/HER2-, WSG-PlanB, and WSG-ADAPT-TN
Source: Breast Cancer Res. 2025 Feb 14;27:22. doi: 10.1186/s13058-025-01969-z (PMC11827153; doi:10.1186/s13058-025-01969-z)
Supplement: Supplementary file 3 — Supplementary Figure 3 [file 13058_2025_1969_MOESM3_ESM.docx]

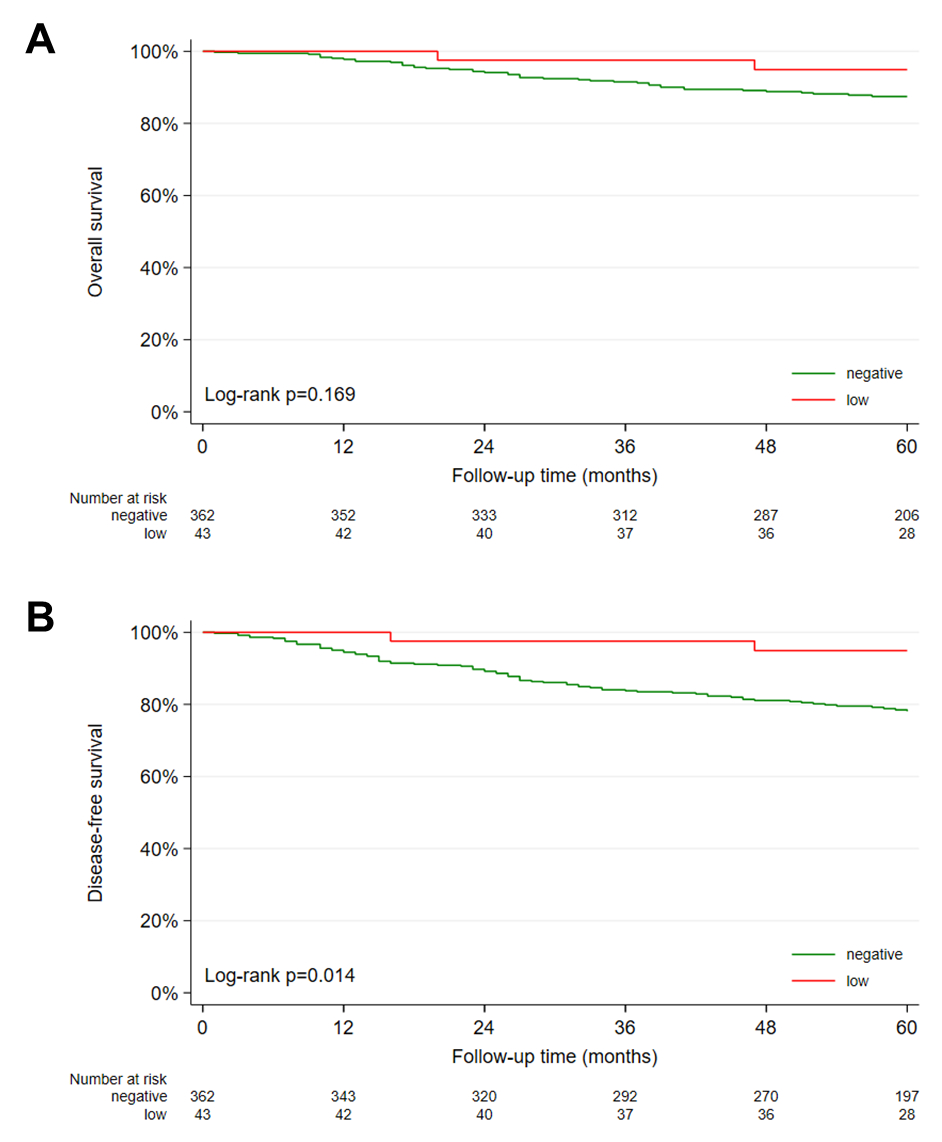


Supplementary Figure 3. Comparison of OS (A) and DFS (B) between HER2-low and HER2-zero tumors in the HR-/HER2- cohort in the WSG-PlanB trial.
