# Supplementary material for: HER2-low status as a distinct breast cancer subtype: myth or truth? Analysis of the WSG trials WSG-ADAPT-HR+/HER2-, WSG-PlanB, and WSG-ADAPT-TN
Source: Breast Cancer Res. 2025 Feb 14;27:22. doi: 10.1186/s13058-025-01969-z (PMC11827153; doi:10.1186/s13058-025-01969-z)
Supplement: Supplementary file 5 — Supplementary Figure 5 [file 13058_2025_1969_MOESM5_ESM.docx]

Supplementary Figure 5. Comparison of iDFS (A, B) and dDFS (C) between HER2-low and HER2-zero tumors at second central assessment in patients treated without (A) and with carboplatin (B), and in cases with pCR in the WSG-ADAPT-HR-/HER2- trial.
